# Network-based piecewise linear regression for QSAR modelling

**DOI:** 10.1007/s10822-019-00228-6

**Published:** 2019-10-18

**Authors:** Jonathan Cardoso-Silva, Lazaros G. Papageorgiou, Sophia Tsoka

**Affiliations:** 1 Department of Informatics, Faculty of Natural and Mathematical Sciences, King’s College London, Bush House, 30 Aldwych, London, WC2B 4BG UK; 2 Centre for Process Systems Engineering, Department of Chemical Engineering, University College London, Roberts Building, Torrington Place, London, WC1E 7JE UK

**Keywords:** QSAR regression, Piecewise linear regression, Mathematical programming, Mixed integer programming

## Abstract

Quantitative Structure-Activity Relationship (QSAR) models are critical in various areas of drug discovery, for example in lead optimisation and virtual screening. Recently, the need for models that are not only predictive but also interpretable has been highlighted. In this paper, a new methodology is proposed to build interpretable QSAR models by combining elements of network analysis and piecewise linear regression. The algorithm presented, modSAR, splits data using a two-step procedure. First, compounds associated with a common target are represented as a network in terms of their structural similarity, revealing modules of similar chemical properties. Second, each module is subdivided into subsets (regions), each of which is modelled by an independent linear equation. Comparative analysis of QSAR models across five data sets of protein inhibitors obtained from ChEMBL is reported and it is shown that modSAR offers similar predictive accuracy to popular algorithms, such as Random Forest and Support Vector Machine. Moreover, we show that models built by modSAR are interpretatable, capable of evaluating the applicability domain of the compounds and serve well tasks such as virtual screening and the development of new drug leads.

**Electronic supplementary material:**

The online version of this article (10.1007/s10822-019-00228-6) contains supplementary material, which is available to authorized users.

## Introduction

Quantitative Structure-Activity Relationship (QSAR) methods employ the molecular properties of chemical compounds to model biological activity against a target [1]. Such drug discovery can draw hypotheses from data, facilitate understanding of drug action mechanisms, allow virtual screening for molecules that have not yet been tested against a target of interest [2], optimise strategies for developing new drugs from a series of potentially suitable compounds [3] or re-purpose existing medicines to different treatments [4]. QSAR models involve representation of each input compound as a set of numerical values (descriptors), before an algorithm is applied to predict the biological activity of each compound against a target on the basis of patterns identified in the data.

Algorithms used for QSAR models range in terms of interpretability. Traditional methods such as linear regression [5] are mathematically descriptive, result in models that provide insight to prediction through appropriate rules and thereby can reveal the most informative molecular properties in the dataset. In other methods (for example, ensemble methods Random Forest [6] and Extreme Gradient Boosting [7], the modern architectures of artificial neural networks in Deep Learning [8], or in consensus of various machine learning techniques [9]), higher prediction accuracy is achieved at the expense of model interpretability. In this later case, it is difficult or even impossible to trace a relation between molecular descriptors and biological activity in a mechanistically descriptive or interpretable means [10].

Piecewise linear regression algorithms applied in QSAR studies are mathematically descriptive, generate interpretable models in the form of a numerical estimate for the contribution of a descriptor in the activity of target binding, and have been shown to yield competitive predictive performance in various QSAR datasets [11, 12]. Such methodologies rely on identification of optimal splits of the data into subgroups (regions) through a molecular descriptor in the data set, and return a linear equation for each of the regions identified to link molecular descriptors to the biological activity of samples in that region. However, this process may result in groups of molecules with rather dissimilar structures, hindering the search for new chemical compounds. Therefore, aiming to represent groupings of molecules as part of the modelling process, we propose a hybrid methodology that represents molecules as a network according to chemical similarity, groups molecules in modules of similar structure through network community detection and then performs piecewise linear regression in each module.

Network analysis constitutes a well-established component of several fields of research in engineering, social sciences, ecology, as well as biological science [13–18]. Recently, molecular networks (or chemical space networks) have gained attention in the drug discovery community [19], for example in identifying previously unknown drug side effects, mechanisms of action of drugs and also as a visual aid to elucidate structure-activity relationships [20–23]. Cluster analysis of these networks can reveal strategies for repositioning existing drugs to different diseases [24] or the existence of activity cliffs [19].

In this article, an algorithm is presented (modSAR) where a molecular network of protein inhibitors is used and clusters of compounds with similar properties are identified as an integral part of the modelling process. Then, the algorithm employs a mixed integer programming model that determines the optimal split of each cluster into appropriate regions [12]. This way, the algorithm exposes a modular organisation of chemical compounds that is not only useful for an exploratory analysis of the data, but also in turn represents a predictive QSAR model that offers a descriptive basis for searching and designing appropriate chemical compounds, as illustrated in the following sections.

## Methods

### The modSAR algorithm

**Fig. 1:**
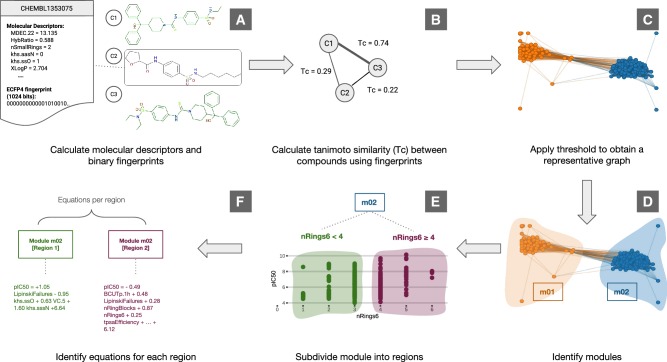
Steps involved in modSAR algorithm

A new method, modSAR, is presented to build QSAR models employing the network representation of chemical compound similarity (Fig. 1). Unlike other studies where a clustering technique is used to partition the data before a QSAR model is constructed [1], module detection is an integral part of modSAR. To create the network, a binary representation of the molecules is obtained with the Extended Connectivity Fingerprint 4 (ECFP4) fingerprint technique (Fig. 1a). The Tanimoto coefficient (Tc) is then used to measure the similarity between molecules (Fig. 1b) and an optimal threshold is applied so that only pairs of molecules with a significant similarity are represented (Fig. 1c). Once the network is created, modSAR applies community detection to indicate clusters of molecules with similar chemical structure (Fig. 1d). Finally, each community is in turn used as input to a combinatorial optimisation model for piecewise linear regression, where a feature is determined to best separate the data into regions (Fig. 1e) and linear equations are identified to predict the outcome variable in each region (Fig. 1f) [12].

Overall, input molecules are subdivided according to their chemical similarity, before the development of linear equations to describe the biological activity of subgroups via combinatorial optimisation, which can describe the relationship between molecular descriptors and biological activity. Each of the steps in this strategy—network creation, SAR modelling and predictions of biological activity for new molecules—are described in the sections below.

### Data

Five data sets were obtained from the ChEMBL database, using the same endpoints used to benchmark algorithms in [25] and [12]. Each data set contains a list of chemical compounds along with their respective $IC_{50}$ value against a common target [26]. To perform regression analysis, the log value of $IC_{50}$ is more frequently used and is defined as $pIC{50} =\,-\,log_{10}(IC_{50})$ (PCHEMBL_VALUE column in ChEMBL). An updated list of the chemical compounds for each dataset was downloaded from ChEMBL. Compounds with dubious measurements (DATA_VALIDITY_COMMENT column in ChEMBL) were removed. In cases of multiple entries for a compound, if relevant activities showed standard deviation greater than 1, $sd_{IC_{50}} > 1$, compounds were removed from the data set, otherwise one entry for the compound was kept with activity equal to the median of the multiple entries.

The Java Chemistry Development Kit (CDK) version 1.5.13 [27] and its R interface [28] was used to calculate 1D and 2D molecular descriptors for the compounds, providing 200+ numerical values to describe each structure (Fig. 1a). These features were processed and scaled from 0 to 1 following the procedure described in Tsiliki et al. 2015 [29]. Finally, highly correlated descriptors and those with a near zero variance in their distributions were removed from the data set using the R package *caret* [30]. A description of data sets after preprocessing is given in Table 1.

**Table 1:** Data sets used in this study

Data Set	Biological endpoint	Source	Samples	Descriptors
hDHFR	Human dihydrofolate reductase	CHEMBL202	542	76
rDHFR	Rat dihydrofolate reductase	CHEMBL2363	875	80
CHRM3	Human muscarinic acetylcholine receptor M3	CHEMBL245	588	87
NPYR1	Human neuropeptide Y receptor type 1	CHEMBL4777	354	70
NPYR2	Human neuropeptide Y receptor type 2	CHEMBL4018	374	67

### Network construction and SAR modelling

To represent molecules as a network, a metric of similarity between chemical compounds is required. A common strategy is to represent each molecule as a fingerprint, i.e. a sequence of binary digits, and apply the Tanimoto coefficient (Tc) [31], which reflects the degree of similarity between compound pairs as a numerical value between 0 and 1 (Fig. 1b). Among the most common fingerprint techniques, the class of Extended Connectivity Fingerprint (ECP) [32] was chosen, where the molecule is represented as a graph and for any given atom both the bonds in that atom and the bonds in its vicinity are used to generate a set of numerical values. Once every atom in the graph has been characterised in this way, a hash function is applied to convert all numerical values to a vector of binary numbers. Here, one of the most popular configurations of this type of fingerprint technique (ECFP4) is applied, where the neighbourhood of up to four degrees of separation of each atom is considered and the size of the binary string is fixed to 1024 bits (Fig. 1a).

After calculating the similarity between compounds, a threshold is defined so that only structures with a significant similarity are linked in the network. There is no consensus on the most appropriate threshold, as the selection of the value depends on the application and on the fingerprint used [33]. For networks built with ECFP4, $t_\alpha = 0.30$ is typically applied so as to represent remote structural similarity [22, 34] and values around 0.50 or 0.60 are reported in the literature in cases where the most similar compounds are linked [35, 36]. The threshold value can also be set to achieve a desired edge density on the network [23].

Recently, Zahoránszky-Kőhalmi et al. [37] have demonstrated that an optimal threshold value, $t^*_{\alpha }$ can be found and corresponds to a peak in the average clustering coefficient (ACC) of the network. When $t_\alpha = 0$, all nodes are connected to each other and the network has maximum ACC ($ACC = 1$). However, by continually increasing the threshold value, ACC tends to decline initially, until increasing again to a peak generally in the interval $0.20< t_{\alpha } < 0.40$. ACC declines with higher threshold values and, although some other value peaks may be observed, none of them leads to a higher ACC than the initial one. The authors have also observed that the most coherent groupings of molecules, as detected by a community detection algorithm, were found when threshold values were close to the initial peak. In our study, similar effect was obtained for ACC and therefore the methodology by Zahoránszky-Kőhalmi et al. was used to define the network threshold for each dataset (Fig. 1c).

After construction of the molecular similarity graph, modSAR detects modules of chemical compounds with similar structure using the Louvain method [38], a fast community detection algorithm that optimises the modularity metric, *Q*, in complex networks [39] (Fig. 1d). In the following step, each of the modules detected are used as input to the Optimal Piecewise Linear Regression Algorithm with Regularisation (OPLRAreg) [12], so as to generate separate QSAR models for each community. OPLRAreg splits molecules within each community into subgroups, in this case defined as regions. Here, each molecule is represented by molecular descriptors, numerical values representing characteristic structures of the compounds, such as the amount of fragments and specific functional groups found in the molecule, as well as experimental measures such as logP or even abstract attributes extracted from the graph of the chemical compound itself. Through the use of these descriptors, OPLRAreg identifies the one that best separates the molecules into regions, before each region is modelled by an optimal linear equation (Fig. 1e, f).

In cases of increased heterogeneity in the dataset, the community detection step may reveal communities consisting of a single member molecule. Such singleton modules can sometimes be considered as structural outliers and removed from QSAR models [1], however here singletons are included, as they do not interfere with prediction arising via other modules.

### Prediction of new samples

After training, modSAR proceeds by choosing which of the various QSAR sub-models to use to predict the activity of a new molecule that was not part of the training set. To determine the neighbourhood of a test sample $s_{test}$, the similarity of $s_{test}$ to all samples in the trained graph is calculated and the module where $s_{test}$ can be allocated is determined according to one of possible three cases: (i) In the simplest case, where the test sample has many connections to trained data, $s_{test}$ is assigned to the module to which it has more connections above the threshold $t_\alpha$. (ii) If the test sample is connected to multiple modules, $s_{test}$ is assigned to the module corresponding to the largest average similarity. (iii) In cases where the test sample does not have any neighbours in the graph, $s_{test}$ is assigned to the module of its most similar compound in the graph. Note, however, that predictions in this last case may not be reliable, since $s_{test}$ is dissimilar to samples in the trained data and it can be considered to be outside the applicability domain (AD) of the QSAR model.

### Implementation details and algorithm validation

**Fig. 2:**
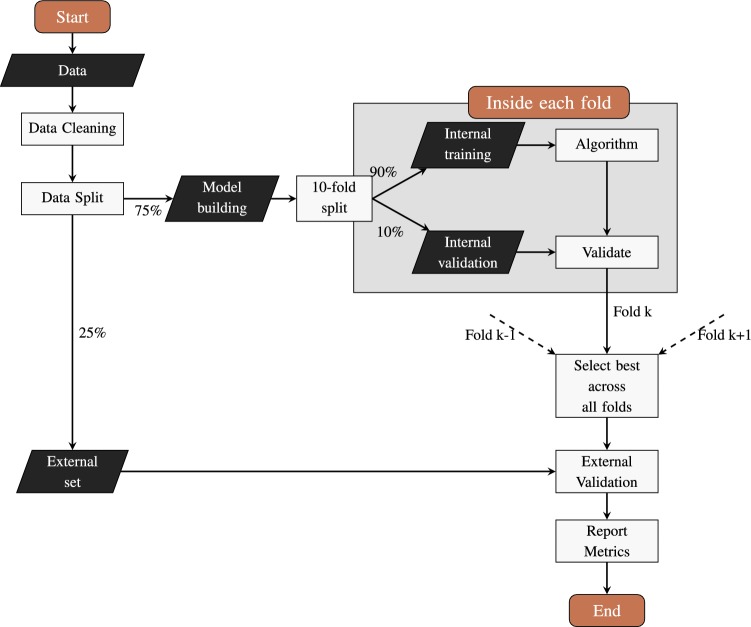
Validation scheme adopted in this study

The validation scheme used in this study is illustrated in Fig. 2 and is aligned with state of the art QSAR model validation procedures [1, 29]. Data sets are initially split randomly, $75\%$ of each data set is used for model building while remaining $25\%$ is used only as external validation set. The subset used for model building ($75\%$) is again divided into 10 training and test folds repeated 10 times, as per function *createMultiFolds* from *caret* package [30] . We note that the optimal threshold value $t_{\alpha }^*$ is identified using only samples in the model building set ($75\%$), the external set is not used while training the algorithm. This threshold is then used to construct the networks at every training fold.

Sensitivity analysis for the regularisation parameter $\lambda$ was performed ( $\lambda \in \{0.005, 0.05, 0.10\}$) and a value of 0.005 was used in all examples. Therefore, through this cross-validation strategy, each algorithm produces 100 different QSAR models constructed from different subsets of data. Of these, the model that had the smallest mean absolute error (MAE) is selected and used to predict biological activity of samples in the external validation set ($25\%$). This procedure of model selection was repeated five times for each data set. Molecular descriptors and ECFP4 fingerprints were computed using Chemistry Development Kit (CDK) [27] implemented in the R package rcdk [28]. A list with the molecular descriptors used in this study can be seen in the Supplementary Material. The algorithm and the cross-validation procedure was implemented in Python while mathematical programming models in OPLRAreg were developed with Pyomo version 5.2 [40] and solved with CPLEX MIP solver [41].

## Results and discussion

In this section, we discuss the details of QSAR models produced by modSAR. We illustrate the properties of networks captured by the method along with the regression models generated for each module in the network. A description of the overall performance in internal validation and external validation sets of the examples is presented, followed by a discussion about the robustness of modules found during cross-validation. Comprehensive comparison with state of the art machine learning algorithms is also discussed.

### Network properties

Optimal threshold values for each data set were determined following the methodology in Zahoránszky-Kőhalmi et al. [37]. In a preliminary analysis, 100 networks were built by varying threshold values from $t_\alpha = 0$ to $t_\alpha = 1$ in increments of 0.01 for each data set, in which we observed the peak in average clustering coefficient (ACC) as described in that study. Optimal threshold values ($t_{\alpha }^*$) were found within the interval $t_{\alpha }^* \in [0.20, 0.40]$ and the corresponding networks showed a modular structure, with a average clustering coefficients over 0.60 and high modularity values $Q > 0.50$. The relationship between thresholds and average clustering coefficient for each data set in this preliminary analysis can be seen on Fig. S1.

Following the validation procedure represented in Fig. 2, optimal thresholds were identified using only the subsample of data designated for model building at every data split. Table 2 shows the average optimal threshold values for each data set, which varied only slightly, as well as the average clustering coefficient and number of modules and singletons in the networks created by modSAR. Networks of NPYR inhibitors had the lowest threshold values and the greatest number of singleton nodes compared to the other data sets while DHFR data sets had larger thresholds, fewer meaningful modules and much less singletons. The presence of many singletons in the network indicate that many compounds are not similar to any other chemicals in the data set above the threshold value $t_\alpha ^*$ and are therefore disconnected from the networks, indicating that NPYR data sets are more heterogeneous than DHFR examples selected in this study.

**Table 2:** Network properties of network models averaged across all five data splits

Data set	Optimal threshold ($t^*_{\alpha }$)	Avg. clustering coefficient	Number of main modules	Number of singletons
NPYR1	$0.25~ (\pm ~0.02)$	$0.64~ (\pm ~0.01)$	$13.60~ (\pm ~3.78)$	$32.00 ~(\pm ~14.28)$
NPYR2	$0.26~ (\pm ~0.01)$	$0.69~ (\pm ~0.02)$	$10.40~ (\pm ~2.70)$	$25.80~ (\pm ~9.83)$
CHRM3	$0.31~ (\pm ~0.00)$	$0.73~ (\pm ~0.01)$	$16.60~ (\pm ~1.67)$	$23.80~ (\pm ~3.03)$
hDHFR	$0.35~ (\pm ~0.01)$	$0.75~ (\pm ~0.01)$	$11.40~ (\pm ~0.55)$	$5.60~ (\pm ~1.34)$
rDHFR	$0.38~ (\pm ~0.01)$	$0.64~ (\pm ~0.00)$	$8.40~ (\pm ~1.67)$	$2.20~ (\pm ~0.84)$

Note that modSAR models take this heterogeneity into account during the training stage of the algorithm. Because each singleton is in a module of its own, no molecular descriptor information is used and the QSAR model of this compound is simply given by its activity value. If modSAR is used to predict the activity of a very similar molecule, the algorithm will return the same IC_50_ value of the singleton.

### Output of modSAR models

**Fig. 3:**
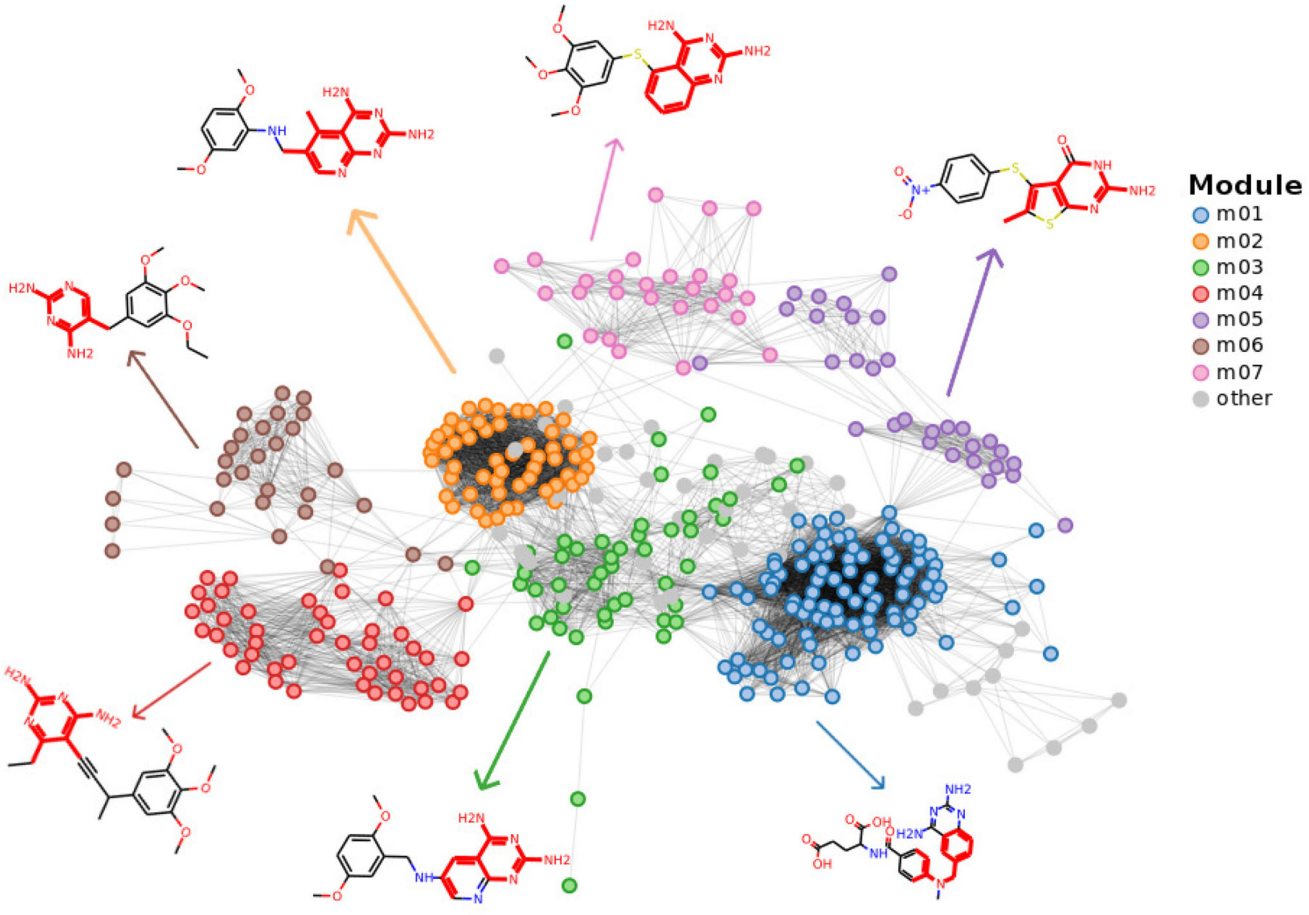
Visualisation of the hDHFR network. Edges represent compounds with similarity above the threshold value $t^*_{\alpha } = 0.29$. A representative compound and the maximum common substructure (in red) for each module is indicated

Here, we illustrate the application of modSAR in building a QSAR model for hDHFR inhibitors, and discuss the interpretation of the model in its entirety and the efficacy of the method for drug discovery. In this example, the optimal threshold value for hDHFR data set was $t_\alpha ^* = 0.29$ and therefore all pairs of molecules with a similarity above this threshold are connected in the network. In Fig. 3, the network for this dataset and the seven large modules as identified by modSAR at the first clustering level are shown. To facilitate understanding of this initial grouping, we show the most representative compound in each module, determined as the one with the largest number of neighbours. The fragments highlighted in red indicate the maximum common substructure (MCS) shared by all compounds in the module.

The networks were drawn using force-directed graph visualisation algorithm Fruchterman Reingold [42], so compounds with similar sub-structures tend to be positioned close together in the network layout. In this type of network visualisation, similar modules also tend to be clustered together. For example, modules m02 and m03 are interconnected by many links and this high connectivity reflects their similar representative structures. However, each module has a unique MCS and closer inspection of the modules would reveal that compounds are more similar to other nodes in the same cluster than to those in neighbouring modules. These properties of network clusters can guide a computer-aided search for promising compounds, e.g. through scaffold hopping analysis using the core of each module to search for new molecules that are either more potent or capable of improving ADMET profile [43]. It is also noted that granularity of clustering can be changed, in cases where the compound similarity in modules needs to be adjusted [44].

At the second level of clustering, combinatorial optimisation through OPLRAreg is used to identify a single molecular descriptor optimal in subdividing each module into regions. Initially, the algorithm finds two subdivisions of the data, in order to fit an optimal relation between the descriptors and the predicted variable ($pIC_{50}$) and then iteratively increases the number of divisions until there is no improvement in the fitting error [12]. Table 3 shows the optimal regions, decision rules and equations associated with the major modules in Fig. 3.

**Table 3:** Equations and breakpoints identified by the algorithm for modules indicated in Fig. 3

Module	Region	Rule	Equation	Descriptor distribution
m01	01	$\text {MDEN.11} \le 0.097$	$pIC_{50} =\,-\,0.24\,\text {C1SP3}\,-\,1.31\,\text {FMF} + 0.64\,\text {LipinskiFailures}\,-\,1.98\,\text {MDEN.22}\,-\,0.65\,\text {SCH.5}\,-\,1.28\,\text {khs.aaO} +1.38\,\text {khs.aaS}\,+\,0.84\,\text {khs.sCl}\,-\,0.26\,\text {khs.ssS}\,-\,0.26\,\text {khs.sssN}\,-\,1.47\,\text {khs.tsC}\,+\,2.65\,\text {tpsaEfficiency.1}\,+\,5.19$	
	02	$\text {MDEN.11} > 0.097$	$pIC_{50} =\,-\,0.72\,\text {ATSm1}\,+\,0.30\,\text {C2SP2}\,-\,0.29\,\text {LipinskiFailures}\,-\,0.41\,\text {MDEN.22}\,-\,0.89\,\text {SCH.5}\,-\,2.64\,\text {khs.aaO}\,+\,1.72\,\text {khs.sCl}\,-\,6.95\,\text {khs.ssCH2}\,-\,1.57 \,\text {khs.ssS}\,+\,0.07\,\text {khs.sssN}\,+\,9.78$	
m02	01	$\text {khs.ssCH2} \le 1.13$	$pIC_{50} =\,-\,0.62\,\text {MDEC.12}\,+\,1.87\,\text {VC.5}\,+\,0.44\,\text {khs.aaaC}\,-\,1.65\,\text {khs.ssS}\,-\,1.71\,\text {khs.sssN} + 1.10\,\text {khs.ssssC} + 7.66$	
	02	$\text {khs.ssCH2} {>} 1.13$	$pIC_{50} =\,+\,0.21\,\text {FMF}\,-\,0.53\,\text {khs.ssO} + 0.03\,\text {khs.sssN} + 7.23\,\text {khs.ssssC} + 5.96$	
m03	01	$\text {khs.sssN} \le 0.98$	$pIC_{50} =\,+\,1.63\,\text {LipinskiFailures}\,-\,0.61\,\text {MDEC.12} + 0.27\,\text {MDEC.33}\,-\,2.55\,\text {MDEN.22}\,-\,0.50\,\text {SCH.5} + 0.44\,\text {khs.aaCH} + 0.07\,\text {khs.aaS} + 0.05\,\text {khs.aaaC} + 0.29\,\text {nAtomP} + 6.41$	
	02	$\text {khs.sssN} > 0.98$	$pIC_{50} =\,-\,3.62\,\,\text {C1SP3} + 2.13\,\text {LipinskiFailures} + 1.03\,\text {MDEC.12}\,-\,0.96\,\text {MDEC.33}\,-\,8.03\,\text {MDEN.22} + 2.24\,\text {khs.sCl} + 7.88\,\text {nAtomP} + 4.81$	
m04	01	$\text {C1SP3} \le 2.85$	$pIC_{50} =\,+\,0.22\,\text {C3SP3} + 1.56\,\text {MDEC.12} + 0.30\,\text {MDEC.23} + 1.27\,\text {MDEN.22}\,-\,0.05\,\text {MDEO.22} + 0.12\,\text {khs.aaN} + 1.21\,\text {khs.aaaC} + 4.97\,\text {khs.ssCH2}\,-\,0.09\,\text {khs.tsC} + 5.18$	
	02	$\text {C1SP3} > 2.85$	$pIC_{50} =\,-\,0.75\,\text {C3SP2}\,-\,1.80\,\text {MDEC.12} + 0.69\,\text {MDEO.22} + 0.64\,\text {khs.ssCH2} + 7.40$	
m05	01	$\text {MLogP} \le 0.46$	$pIC_{50} =\,+\,6.25$	
	02	$0.46 \le \text {MLogP} \le 0.96$	$pIC_{50} =\,+\, 0.40\,\text {ATSm1}\,-\,0.31\,\text {FMF}\,-\,1.13\,\text {LipinskiFailures} + 1.00\,\text {khs.aaS} + 0.02\,\text {khs.sCl} + 1.33\,\text {khs.sssN} + 3.77\,\text {nAtomLAC} + 4.82$	
	03	$0.96 \le \text {MLogP} \le 1.13$	$pIC_{50} =\,-\, 1.87\,\text {MDEN.11} + 0.53\,\text {khs.aaS} + 5.55$	
	04	$1.13 \le \text {MLogP} \le 1.29$	$pIC_{50} =\,-\, 0.12\,\text {nAtomLAC} + 4.52$	
	05	$\text {MLogP} > 1.29$	$pIC_{50} =\,+\,0.49\,\text {MDEC.23} + 0.12\,\text {khs.aaS} + 0.41\,\text {nAtomLAC} + 5.25$	
m06	01	$\text {khs.aaCH} \le 3.18$	$pIC_{50} =\,-\,0.50\,\text {C1SP3} + 0.59\,\text {C3SP3} + 0.22\,\text {MDEC.33}\,-\,1.76\,\text {VCH.6} + 6.73\,\text {VP.7} + 2.97\,\text {khs.dsCH}\,-\,0.31\,\text {khs.sssN}\,-\,1.09\,\text {khs.tsC} + 6.08$	
	02	$3.18 \le \text {khs.aaCH} \le 4.18$	$pIC_{50} =\,+\, 3.91\,\text {MDEC.12} + 0.02\,\text {khs.aaaC}\,-\,0.18\,\text {khs.tsC} + 4.49$	
	03	$\text {khs.aaCH} > 4.18$	$pIC_{50} =\,+\,0.78\,\text {MDEC.33} + 1.16\,\text {VCH.6} + 2.04\,\text {khs.aaNH} + 3.25\,\text {khs.aaaC} + 5.29$	
m07	01	$\text {tpsaEfficiency} \le 0.30$	$pIC_{50} =\,+\,15.24\,\text {MDEC.12}\,-\,6.65\,\text {khs.ssS}\,-\,1.42\,\text {khs.sssN} + 4.97$	
	02	$0.30 \le \text {tpsaEfficiency} \le 0.38$	$pIC_{50} =\,-\,0.15\,\text {C1SP3} + 1.37\,\text {MDEC.12}\,-\,0.51\,\text {MDEN.11}\,-\,0.55\,\text {MDEO.22}\,-\,1.71\,\text {khs.ssS}\,-\,0.27\,\text {khs.ssssC}\,-\,1.24\,\text {khs.tsC}\,-\,12.80\,\text {nAtomLC} + 9.35$	
	03	$0.38 \le \text {tpsaEfficiency} \le 0.46$	$pIC_{50} =\,-\,0.91\,\text {MDEN.11}\,-\,6.15\,\text {khs.tsC} + 7.13$	
	04	$\text {tpsaEfficiency} > 0.46$	$pIC_{50} =\,+\,4.62$	

Table 3 also includes graphs to show the distribution of the descriptor that generates the regions in each module, coloured according to the region in which the samples lie. Note that the molecular descriptors vary substantially from equation to equation since the algorithm also incorporates an implicit selection of variables. Among attributes (more than 200) calculated for each molecule in the data set, the optimisation and regularisation process employed in the OPLRAreg algorithm generates a minimum set of features to build the regression model. These descriptors range from those representing distances between specific atoms (e.g. MDEN11, the average distance between primary nitrogen atoms) to counts of specific fragments (e.g. Kier Hall descriptors $khs-*$) or features representing molecular lipophilicity, such as the Manhold method for logP estimation (MLogP). We note that in the case of whole-molecule descriptors, direct interpretation and even more so targeted molecule de novo design can be challenging.

The characteristics of these optimal descriptors might also serve to support better targeting in QSAR studies. For example, in the case of hDHFR inhibitors if one is interested in studying potent molecules with the common core identified in module m05 and greater logP, one can focus and screen samples within region 05. The equation predicts that potency in this group of molecules is positively associated with the increase in average distance between secondary and tertiary carbons (MDEC.23), the number of occurrences of a fragment identified by khs.aaS descriptor (SMARTS: [S,sD2H0](:*):*) as well as the number of atoms in the largest aliphatic chain (nAtomLAC). Such insight can pave the way for investigating the characteristics of molecules in this region of molecular space further.

**Fig. 4:**
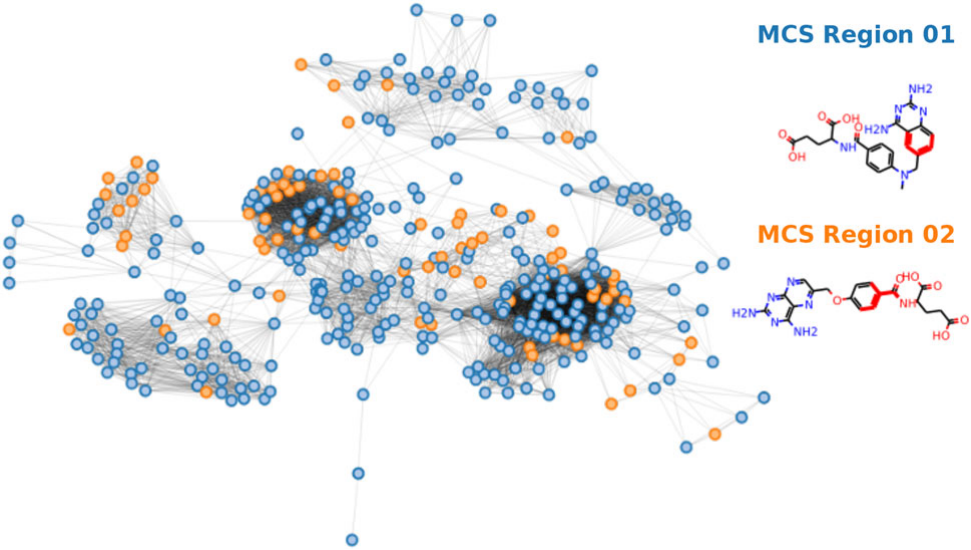
Regions identified by OPLRAreg for hDHFR data set

It is important to note that if the piecewise linear regression step was applied directly to the entire data set, without the initial clustering step, the biological activity could be predicted with reasonable prediction accuracy [12]. However, by adding a first level of clustering, modSAR yields more descriptive and informative clusters since it finds patterns in the data that are linked to compound structural similarity. For example, applying piecewise linear regression directly to the hDHFR data set without the community detection step, compounds are separated into two regions depending on whether the molecule contains a fragment identified by the molecular descriptor $khs-ssS$, region 01 and region 02, respectively (Fig. 4). Compounds in the same group do not share structural similarity other than the fragment indicated and even the MCS of each module is rather non descriptive. Comparing Figs. 4 to 3, it is observed that in the former case the most representative molecule is less meaningful as modules members are diverse and contain dissimilar molecular structures.

### Overall performance and comparative results

The performance of modSAR across the external validation set is summarised in Table 4, where mean absolute error (MAE) and standard deviation (SD) of prediction errors are represented for each of the five datasets, as per the validation procedure described in "Methods" section. The table separates the results of samples inside the applicability domain (AD) from those outside AD. In terms of predictive performance, the predictions made by modSAR in the external validation set were close to $\pm 0.60$ log units for NPYR1, NPYR2 and rDHFR data sets and $\approx \pm 0.70$ log units for CHRM3 and hDHFR.

**Table 4:** Average performance of modSAR in the external set ($MAE \pm SD$)

Data split	Data set				
	NPYR1	NPYR2	CHRM3	hDHFR	rDHFR
Within applicability domain					
1	$0.64~ (\pm ~0.63)$	$0.46~ (\pm ~0.42)$	$0.69~ (\pm ~0.55)$	$0.78~ (\pm ~0.62)$	$0.56~ (\pm ~0.53)$
2	$0.60~ (\pm ~0.71)$	$0.48~ (\pm ~0.37)$	$0.63~ (\pm ~0.54)$	$0.78~ (\pm ~0.81)$	$0.58~ (\pm ~0.52)$
3	$0.56~ (\pm ~0.60)$	$0.62~ (\pm ~0.60)$	$0.65~ (\pm ~0.57)$	$0.69~ (\pm ~0.71)$	$0.54~ (\pm ~0.56)$
4	$0.55~ (\pm ~0.62)$	$0.56~ (\pm ~0.50)$	$0.63~ (\pm ~0.59)$	$0.71~ (\pm ~0.62)$	$0.58~ (\pm ~0.60)$
5	$0.74~ (\pm ~0.80)$	$0.62~ (\pm ~0.54)$	$0.61~ (\pm ~0.56)$	$0.73~ (\pm ~0.60)$	$0.57~ (\pm ~0.55)$
Outside applicability domain					
1	$0.50~ (\pm ~0.58)$	$0.25~ (\pm ~0.26)$	$0.62~ (\pm ~0.48)$	$0.45~ (\pm ~0.00)$	–
2	$0.38~ (\pm ~0.52)$	$0.70~ (\pm ~0.32)$	$1.94~ (\pm ~0.78)$	$3.20~ (\pm ~0.99)$	–
3	$0.17~ (\pm ~0.12)$	$0.39~ (\pm ~0.39)$	$1.32~ (\pm ~1.18)$	$0.71~ (\pm ~0.49)$	–
4	$0.27~ (\pm ~0.20)$	$0.78~ (\pm ~1.41)$	$1.08~ (\pm ~1.02)$	$0.45~ (\pm ~0.00)$	$0.92~ (\pm ~0.68)$
5	$0.60~ (\pm ~0.76)$	$0.54~ (\pm ~0.68)$	$1.30~ (\pm ~0.92)$	$1.20~ (\pm ~0.75)$	$0.58~ (\pm ~0.00)$

As expected, there is a lot of variation when predicting compounds that are not well represented in the trained graph (outside AD) since those samples are not within the threshold value of the samples used during cross-validation in the training stage. If a sample is outside AD, modSAR is still able to predict the biological activity with the model of its nearest neighbour but the prediction is less reliable, as described in "Prediction of new samples" section. Indeed, predictions of samples outside AD showed larger errors in CHRM3, rDHFR and reached an mean absolute error as high as 3.20 log units in hDHFR, an error 5 times higher compared to samples that fall within the applicability domain for that data set. However, predictions made outside of AD for NYPR1 and NYPR2 data sets were not as discrepant. Similar to predictions within the applicability domain, the absolute mean error (MAE) for these two datasets was below 0.6 in most cases, with an equally low standard deviation compared to the other data sets.

Results of the internal cross-validation can be seen in Table S2. The accuracy of predictions in the internal validation set is similar to those seen in Table 4 ($MAE \approx 0.60-0.70$), but the average fit error of in the training set is much smaller for data sets NYPR1 and NYPR2 ($MAE \approx 0.24-0.26$) which, in combination with the results described above, might suggest that these datasets are less heterogeneous and easier to model.

**Fig. 5:**
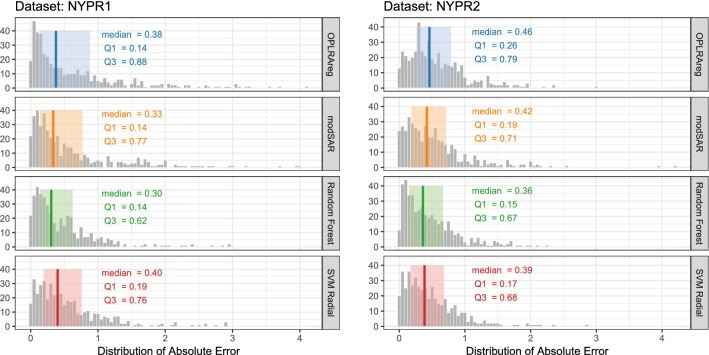
Predictive performance of algorithms for NPYR data sets

**Fig. 6:**
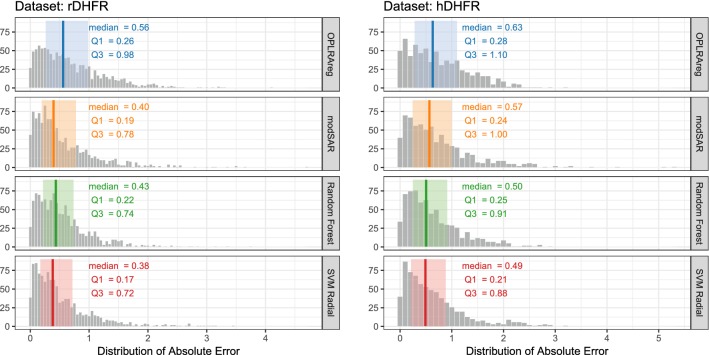
Predictive performance of algorithms for DHFR data sets

**Fig. 7:**
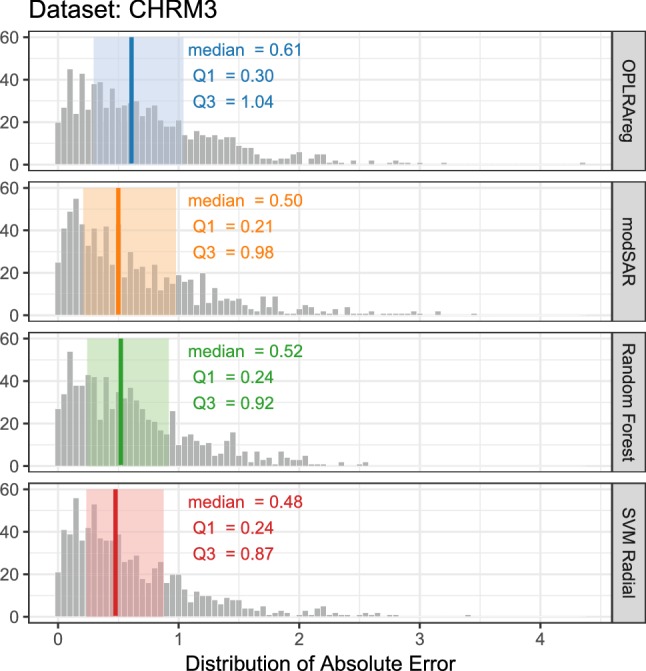
Predictive performance of algorithms for CHRM3 data set

Results obtained through modSAR were compared to regression without the prior stage of community detection (OPLRAreg) [12], as well as popular algorithms (Random Forest [45] and SVM Radial) implemented in the caret package [30]. Figures 5, 6, 7 depict the distribution of absolute prediction errors in the external set of all five data sets. Only predictions made for samples inside the applicability domain, as obtained by modSAR, are shown in the plots. The vertical line indicates the median absolute error while the shaded coloured area depicts interquartile range of predictions made by the algorithms and gives an intuition about the error dispersion of the compared algorithms. As an example, most compounds in the NYPR1 data set are predicted by OPLRAreg within 0.14 to 0.44 log units of accuracy while most predictions made by modSAR vary a little less and range from 0.14 to 0.77 log units (Fig. 5a).

In fact, modSAR predictions had less variability than OPLRAreg in all five examples of this study and the predictions were even closer to the traditional machine learning methods. Median values and interquartile ranges in modSAR predictions were very similar to Random Forest and Support Vector Machine. This again underlines the advantages of a more transparent model like modSAR compared to black-box methods. The proposed method allows investigating the characteristics of the QSAR model directly, without the need for a post-hoc interpretation step.

### Presence of activity cliffs

The impact of activity cliffs in QSAR models with modSAR was investigated. Activity cliffs are discontinuities in structure-activity relationships, whereby molecules with similar structures have a large variation in activity response [36]. Activity cliffs can be measured numerically and molecules classified as “high”, “intermediate” or “low” depending on their activity discontinuity [22]. If a molecule is labelled “high” in activity cliff (AC), most of its neighbours, although structurally similar, have an unexpectedly large difference in biological activity and conversely a low AC implies variance in activity within the neighbourhood of a molecule that is more easily explained by their structural dissimilarity. The proportion of activity cliff classes in these networks is large, as Table 5 shows that more than half of the samples in all examples are classified as either intermediate or high in terms of AC.

**Table 5:** Proportion of activity cliff classes in the QSAR data sets studied

Dataset	Discontinuity class		
	High (%)	Interm. (%)	Low (%)
NPYR1	22.65	35.54	41.81
NPYR2	21.73	33.23	45.05
CHRM3	21.15	30.66	48.20
rDHFR	21.32	34.24	44.44
hDHFR	25.86	28.39	45.75

**Fig. 8:**
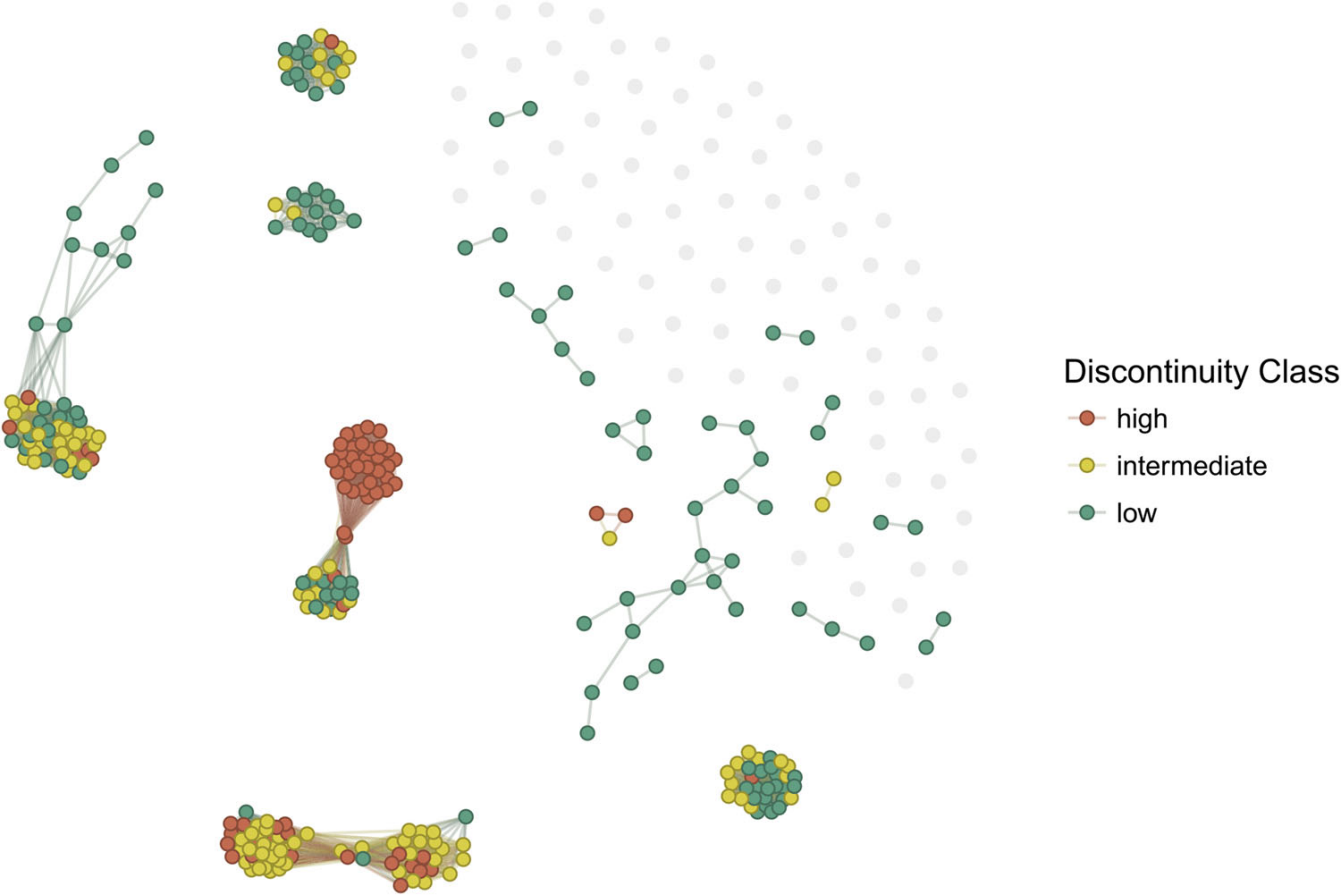
Activity cliffs in NPYR1 network

Activity cliffs are not necessarily distributed evenly across the network. Figure 8, for example, displays the network of NPYR1 inhibitors where each disconnected component corresponds to a module and nodes are coloured according to their discontinuity class. Singletons are represented as grey in the picture since activity cliffs are not defined for these samples. Note that some modules contain most samples in medium or high discontinuity regions. Here,it is noted that the use of modSAR on these modules is able to identify the structural characteristics or molecular descriptors that best explain these discontinuities.

Despite the difficulties of modelling molecules in high discontinuity regions, the modSAR algorithm also exhibited better results in this regard when compared to OPLRAreg. Table 6 shows the average reduction in mean absolute error (MAE) and in the standard deviation (SD) of absolute errors (shown inside brackets) for external set predictions. MAE was smaller for samples in intermediate and low discontinuity class in almost all data sets. MAE is also 33%, 24% and 10% smaller for high AC samples in NPYR1, hDHFR and rDHFR data sets, respectively. modSAR shows improvement in SD in all data sets, which means that the dispersion of errors of all cases was reduced, even those with a modest MAE reduction. Notably, CHRM3 was the only data set for which modSAR made a worse prediction for high AC samples, with $6.40\%$ average increase in MAE, however, the error dispersion was improved considerably, with a reduction of $42.64\%$ in SD.

**Table 6:** Reduction in MAE and SD of errors by modSAR compared to OPLRAreg per discontinuity class

Dataset	Discontinuity class		
	High (%)	Interm. (%)	Low (%)
NPYR1	− 32.87 (− 44.88)	− 8.06 (− 32.44)	− 14.44 (− 8.61)
NPYR2	0.32 (− 14.04)	− 1.61 (− 25.81)	− 8.57 (− 6.62)
CHRM3	6.40 (− 42.64)	− 14.53 (− 46.56)	− 14.72 (− 26.57)
hDHFR	− 23.70 (− 35.19)	− 0.08 (− 3.71)	0.12 (− 3.93)
rDHFR	− 10.23 (− 38.30)	− 8.31 (− 26.62)	− 33.28 (− 44.10)

## Outlook

We illustrate a possible application of modSAR as a flexible and interpretable methodology towards de novo molecular design [46–48]. Typically, de novo algorithms are evolutionary algorithms that combine fragments and functional groups and follow certain imposed constraints to propose structures that maximise biological activity [49, 50]. Any predictive and validated QSAR model can be used to score the activity of these artificial compounds, but modSAR could have a more important role in de novo design as it could be used to generate additional constraints for these algorithms. This concept is illustrated below.

Suppose one is interested in finding more potent inhibitors in the search space around module m06 and region 03 of Figure 3. The maximum common substructure, along with the breakpoints, equations and the neighbourhood of m06 molecules could be combined to provide the following constraints to a de novo technique for new hDHFR inhibitors, as follows.

- *Neighbourhood constraint*: new chemical entities should be within the applicability domain of module m03. A new molecule is only considered feasible if its similarity to at least one of the compounds in the module is above the threshold $t_\alpha ^* = 0.29$,
- *Breakpoint constraint*: new molecules must satisfy the region 03 division rule, $\text {khs.aaCH} > 4.18$. That is, there must be at least five aliphatic carbon atoms to satisfy the criterion represented by the khs.aaCH descriptor, as illustrated in Figure S2.
- *Equation constraint*: the correlates identified by the equation in Region 03 could be used in the design process. For example, the distance between tertiary carbons (MDEC.33) and the count of khs.aaNH and khs.aaaC fragments should be maximised. Similarly, if there were any negative coefficients in the equation, this variable should be minimised in the optimisation process.

Similar constraints could be obtained for all other modules in the network and the combined set of rules could provide a targeted set of optimisation constraints for de novo design. These constraints could potentially reduce the search space of current algorithms, facilitating the generation of synthetically produced new compounds.

## Conclusions

We have shown that the use of network representation in QSAR studies can improve its effectiveness. The method proposed in this paper, modSAR, uses community detection to identify groups of similar molecules before a combinatorial optimisation step can construct predictive and interpretable QSAR models. Visualisation of such models along with the analysis of the common core of molecules in the modules allow for a quick grasp on heterogeneity that may lie in the data set and facilitates understanding of the characteristics in chemical space.

The analysis described here showed that singletons will generally represent a diverse set of molecules and might indicate a less explored part of chemical space, therefore pointing towards new directions for promising drugs or probe candidates. Dense modules, on the other hand, represent groups of molecules where usually a common core can be identified. An automatic workflow such as modSAR can improve the discovery of these subgroups and help to conceptualise these complex relationships more easily.

modSAR facilitates the identification of groups of molecules with activity cliffs, with the potential to help medicinal chemists identify new promising paths for drug discovery more easily. The algorithm could also be used to generate constraints for de novo drug design, contributing to lead optimisation of promising compounds.

Predictions in the external set were not affected when a minimum number of neighbours was enforced and network properties only changed in data sets that had a large number of singletons. The advantage of this approach lies in the reduced number of modules, which might facilitate the visualisation of network modules but at the cost of generating “artificial” links between non-similar compounds. This trade-off should be taken into account when developing a new model and the requirement for the number of neighbours should probably be driven by the needs of specific QSAR projects. In future work, exploring the impact of other network construction techniques, with different fingerprints and similarity metrics, can be investigated in terms of improving performance.

Other immediate extensions of the current work would be to study selectivity, instead of activity of compounds. It might be possible to identify modules with identifying fragments, substructures or descriptors that help to explain why certain compounds have more affinity for a specific receptor, say NPYR2, than others, e.g. NPYR1. modSAR could also be used to study selectivity of compounds for drug targets in specific organisms. One possible application would be to study the activity of inhibitors of DHFR in other organisms (e.g. *Candida albicans*) compared to the human and rat models.

Future work is planned to address criteria for the selection of descriptors in the application of OPRAreg in QSAR studies. In specific, detailed analysis on the impact of particular descriptors (in terms of correlation or other criteria) can be incorporated in the computational procedure to indicate the robustness of prediction and individual feature contributions. Future work can also extend the use of community detection into the realm of consensus clustering, where multiple layers of networks can be used so as to produce clusters that generalise better from a subsample to a full data set [51–53].

## Electronic supplementary material

Below is the link to the electronic supplementary material.

Electronic supplementary material 1 (PDF 226 kb)

## References

[1] Tropsha A (2010). Mol Inform.

[2] Melo-Filho CC, Dantas RF, Braga RC, Neves BJ, Senger MR, Valente WCG, Rezende-Neto JM, Chaves WT, Muratov EN, Paveley RA, Furnham N, Kamentsky L, Carpenter AE, Silva-Junior FP, Andrade CH (2016). J Chem Inf Model.

[3] Gomes MN, Braga RC, Grzelak EM, Neves BJ, Muratov EN, Ma R, Klein LK, Cho S, Oliveira GR, Franzblau SG, Andrade CH (2017). Eur J Med Chem.

[4] Rescifina A, Floresta G, Marrazzo A, Parenti C, Prezzavento O, Nastasi G, Dichiara M, Amata E (2017). Eur J Pharm Sci.

[5] Mitchell JBO (2014). Wiley Interdiscip Rev.

[6] Devinyak OT, Lesyk RB (2016). Curr Comput-Aided Drug Des.

[7] Sheridan RP, Wang WM, Liaw A, Ma J, Gifford EM (2016). J Chem Inf Model.

[8] Uesawa Y (2018). Bioorg Med Chem Lett.

[9] Alves VM, Golbraikh A, Capuzzi SJ, Liu K, Lam WI, Korn DR, Pozefsky D, Andrade CH, Muratov EN, Tropsha A (2018). J Chem Inf Model.

[10] Zhang Q, Li H (2007). IEEE Trans Evolut Comput.

[11] Yang L, Liu S, Tsoka S, Papageorgiou LG (2016). Expert Syst Appl.

[12] Cardoso-Silva J, Papadatos G, Papageorgiou LG, Tsoka S (2019). Mol Inform.

[13] Girvan M, Newman MEJ (2002). PNAS.

[14] Meng F, Wang J, Yang L, Wang S, Jiang W, Li X, Chen X, Lv Y, Wang Z, Li Y (2015). Bioinformatics.

[15] Malod-Dognin N, Gaudelet T, PrŽulj N (2018). Bioinformatics.

[16] Zhou Y, Ni Z, Chen K, Liu H, Chen L, Lian C, Yan L (2013). Protein J.

[17] Hu JX, Thomas CE, Brunak S (2016). Nat Rev Genet.

[18] Danhof M (2016). Eur J Pharm Sci.

[19] Csermely P, Korcsmáros T, Kiss HJM, London G, Nussinov R (2013). Pharmacol Ther.

[20] Boezio B, Audouze K, Ducrot P, Taboureau O (2017). Netw-Based Approach Pharmacol.

[21] Wawer M, Peltason L, Weskamp N, Teckentrup A, Bajorath J (2008). J Med Chem.

[22] Namasivayam V, Gupta-Ostermann D, Balfer J, Heikamp K, Bajorath J (2014). J Chem Inf Model.

[23] Vogt M, Stumpfe D, Maggiora GM, Bajorath J (2016). J Comput-Aided Mol Des.

[24] Udrescu M, Udrescu L (2019). A drug repurposing method based on drug-drug interaction networks and using energy model layouts.

[25] Cortes-Ciriano I, Bender A (2015). J Chem Inf Model.

[26] Brooks HB, Geeganage S, Kahl SD, Montrose C, Sittampalam S, SmithMC, Weidner JR (2012) Basics of enzymatic assays for hts. In: Assay guidance manual. Eli Lilly & Company and the National Center for Advancing Translational Sciences 22553875

[27] Steinbeck C, Han Y, Kuhn S, Horlacher O, Luttmann E, Willighagen E (2003). J Chem Inf Comput Sci.

[28] Guha R (2007). J Stat Softw.

[29] Tsiliki G, Munteanu CR, Seoane JA, Fernandez-Lozano C, Sarimveis H, Willighagen EL (2015). J Cheminform.

[30] Kuhn M (2016) caret: Classification and Regression Training, R package version 6.0-73

[31] Willett P, Barnard JM, Downs GM (1998). J Chem Inf Comput Sci.

[32] Rogers D, Hahn M (2010). J Chem Inf Model.

[33] Stumpfe D, Hu Y, Dimova D, Bajorath J (2014). J Med Chem.

[34] Dimova D, Stumpfe D, Bajorath J (2013). J Chem Inf Mod.

[35] Hu Ye, Stumpfe Dagmar, Bajorath Jürgen (2013). Advancing the activity cliff concept. F1000Research.

[36] Stumpfe D, Bajorath J (2012). J Med Chem.

[37] Zahoránszky-Kőhalmi G, Bologa CG, Oprea TI (2016). J Cheminform.

[38] Blondel VD, Guillaume JL, Lambiotte R, Lefebvre E (2008). J Stat Mech.

[39] Fortunato S, Hric D (2016). Phys Rep.

[40] Hart WE, Laird C, Watson JP, Woodruff DL (2012). Pyomo-optimization modeling in Python, springer optimization and its applications.

[41] Corporation I B M (2009) Ibm ilog cplex v12. 7: User’s manual for cplex

[42] Fruchterman TMJ, Reingold EM (1991). Softw: Pract Exp.

[43] Hu Y, Stumpfe D, Bajorath J (2017). J Med Chem.

[44] Lambiotte R, Delvenne JC, Barahona M (2014). IEEE Trans Netw Sci Eng.

[45] Breiman L (2001). Mach Learn.

[46] Schneider G, Fechner U (2005). Nat Rev Drug Discov.

[47] Schneider G (2013). Drug Discov Today: Technol.

[48] Reker D, Rodrigues T, Schneider P, Schneider G (2014). Proc Natl Acad Sci.

[49] Nicolaou CA, Apostolakis J, Pattichis CS (2009). J Chem Inf Model.

[50] Devi RV, Sathya SS, Coumar MS (2015). Appl Soft Comput.

[51] Bennett L, Kittas A, Muirhead G, Papageorgiou LG, Tsoka S (2015). Sci Rep.

[52] Silva JC, Bennett L, Papageorgiou LG, Tsoka S (2016). Eur Phys J B.

[53] Xu G, Tsoka S, Papageorgiou LG (2007). Eur Phys J B.

