# Supplementary material for: Network-based piecewise linear regression for QSAR modelling
Source: J Comput Aided Mol Des. 2019 Oct 18;33(9):831–44. doi: 10.1007/s10822-019-00228-6 (PMC6825651; doi:10.1007/s10822-019-00228-6)
Supplement: Supplementary file 1 — Electronic supplementary material 1 (PDF 226 kb) [file 10822_2019_228_MOESM1_ESM.pdf]

Supplementary Materials

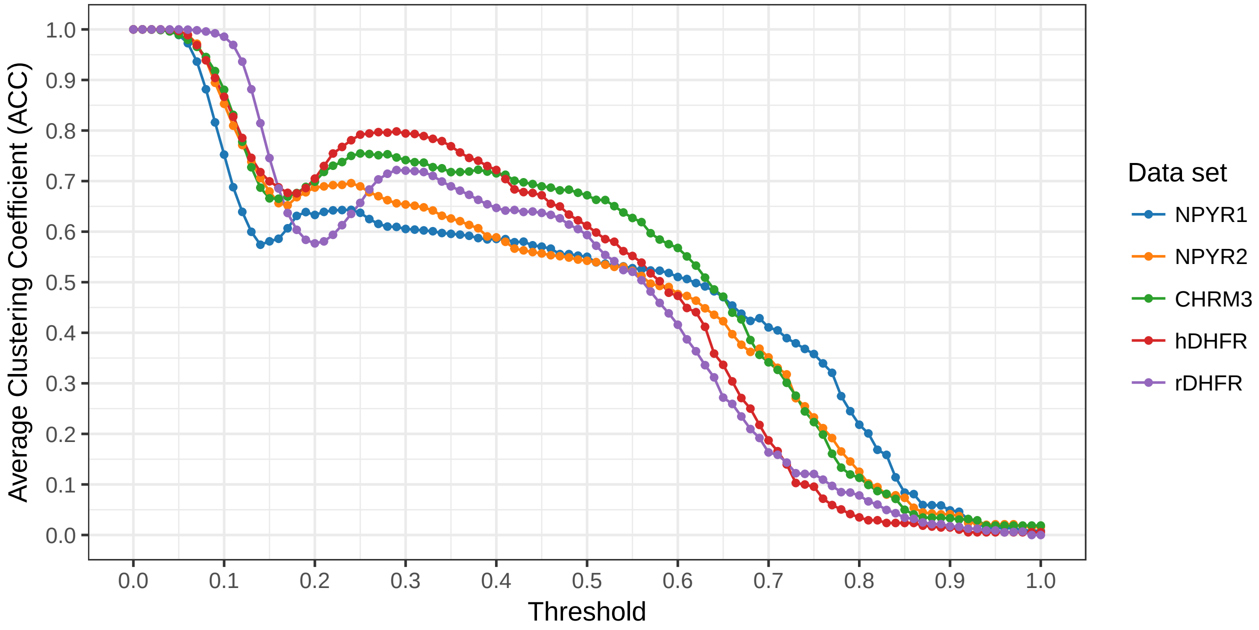

Figure S1: Threshold analysis for network representation of QSAR datasets used in the study

Table S1: Optimal threshold values and network metrics of QSAR data sets

Data set	Nodes	Edge Density	Threshold t^*_α	ACC	Modularity	No. of main modules	No. of singletons	Average shortest path	Average degree	Degree assortativity
NPYR1	247.40 (±0.55)	0.06 (±0.01)	0.25 (±0.02)	0.64 (±0.01)	0.78 (±0.02)	13.60 (±3.78)	32.00 (±14.28)	15.16 (±1.45)	3.95 (±1.32)	0.67 (±0.08)
NPYR2	256.60 (±0.55)	0.13 (±0.01)	0.26 (±0.01)	0.69 (±0.02)	0.60 (±0.03)	10.40 (±2.70)	25.80 (±9.83)	31.97 (±2.18)	2.81 (±0.09)	0.83 (±0.06)
CHRM3	434.80 (±0.84)	0.09 (±0.01)	0.31 (±0.00)	0.73 (±0.01)	0.58 (±0.04)	16.60 (±1.67)	23.80 (±3.03)	40.18 (±2.71)	3.84 (±0.30)	0.67 (±0.02)
hDHFR	377.80 (±0.84)	0.10 (±0.00)	0.35 (±0.01)	0.75 (±0.01)	0.65 (±0.01)	11.40 (±0.55)	5.60 (±1.34)	37.55 (±1.12)	2.81 (±0.05)	0.49 (±0.03)
rDHFR	596.80 (±0.45)	0.08 (±0.01)	0.38 (±0.01)	0.64 (±0.00)	0.65 (±0.02)	8.40 (±1.67)	2.20 (±0.84)	44.80 (±4.40)	2.79 (±0.15)	0.50 (±0.01)

Table S2: Internal training and validation performance of modSAR: (median *MAE* and *MAD* from the median)) under cross-validation

Data Split	Data Set				
	NPYR1	NPYR2	CHRM3	hDHFR	rDHFR
**Internal Training**					
1	0.36 (±0.17)	0.41 (±0.13)	0.51 (±0.06)	0.66 (±0.09)	0.57 (±0.09)
2	0.32 (±0.17)	0.41 (±0.08)	0.54 (±0.06)	0.69 (±0.10)	0.53 (±0.11)
3	0.25 (±0.08)	0.39 (±0.10)	0.53 (±0.05)	0.71 (±0.07)	0.56 (±0.10)
4	0.34 (±0.14)	0.38 (±0.12)	0.55 (±0.09)	0.70 (±0.06)	0.54 (±0.07)
5	0.33 (±0.16)	0.36 (±0.06)	0.54 (±0.05)	0.71 (±0.06)	0.55 (±0.09)
**Internal Validation**					
1	0.73 (±0.11)	0.65 (±0.11)	0.74 (±0.06)	0.81 (±0.07)	0.67 (±0.06)
2	0.68 (±0.11)	0.63 (±0.10)	0.77 (±0.08)	0.83 (±0.06)	0.65 (±0.06)
3	0.63 (±0.05)	0.60 (±0.08)	0.72 (±0.07)	0.83 (±0.07)	0.67 (±0.06)
4	0.72 (±0.12)	0.62 (±0.08)	0.77 (±0.07)	0.84 (±0.07)	0.66 (±0.07)
5	0.69 (±0.12)	0.61 (±0.08)	0.76 (±0.08)	0.85 (±0.07)	0.66 (±0.06)

Table S3: Internal training and validation performance of modSAR: (median *RMSE* and *MAD* from the median) under cross-validation

Data Split	Data Set				
	NPYR1	NPYR2	CHRM3	hDHFR	rDHFR
**Internal Training**					
1	0.68 (±0.21)	0.62 (±0.17)	0.77 (±0.05)	0.92 (±0.07)	0.79 (±0.09)
2	0.57 (±0.29)	0.63 (±0.09)	0.81 (±0.05)	0.94 (±0.08)	0.76 (±0.11)
3	0.48 (±0.24)	0.58 (±0.14)	0.79 (±0.04)	0.97 (±0.03)	0.79 (±0.09)
4	0.64 (±0.20)	0.59 (±0.16)	0.82 (±0.08)	0.96 (±0.03)	0.78 (±0.07)
5	0.62 (±0.22)	0.57 (±0.05)	0.80 (±0.06)	0.98 (±0.03)	0.78 (±0.08)
**Internal Validation**					
1	0.75 (±0.09)	0.64 (±0.07)	0.89 (±0.07)	0.86 (±0.06)	0.78 (±0.08)
2	0.72 (±0.08)	0.63 (±0.08)	0.92 (±0.08)	0.86 (±0.07)	0.78 (±0.06)
3	0.71 (±0.08)	0.63 (±0.08)	0.87 (±0.07)	0.89 (±0.09)	0.78 (±0.06)
4	0.73 (±0.09)	0.67 (±0.06)	0.90 (±0.06)	0.89 (±0.08)	0.78 (±0.07)
5	0.74 (±0.11)	0.61 (±0.07)	0.88 (±0.07)	0.87 (±0.07)	0.80 (±0.07)

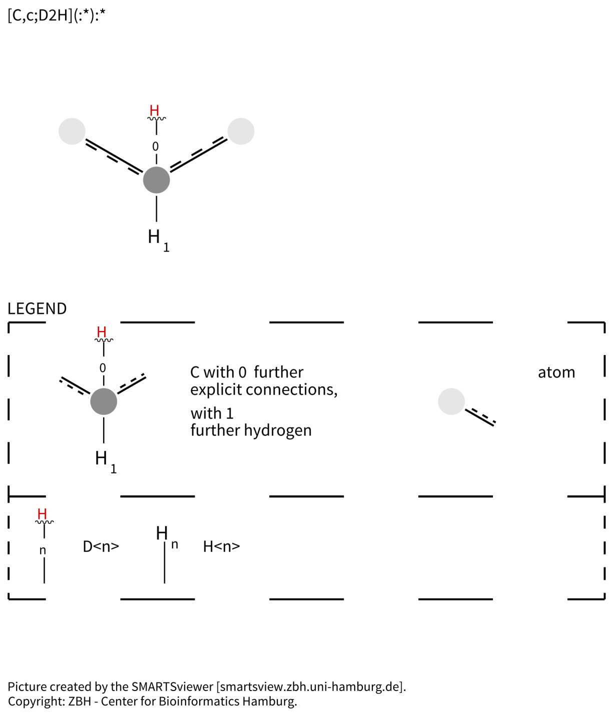

Figure S2: Fragment represented by descriptor khs.aaCH
